# CD73 regulates zoledronate-induced lymphocyte infiltration in triple-negative breast cancer tumors and lung metastases

**DOI:** 10.3389/fimmu.2023.1179022

**Published:** 2023-07-18

**Authors:** Nataliia Petruk, Arafat Siddiqui, Sina Tadayon, Jorma Määttä, Pieta K. Mattila, Arja Jukkola, Jouko Sandholm, Katri S. Selander

**Affiliations:** ^1^ Institute of Biomedicine, University of Turku, Turku, Finland; ^2^ Western Cancer Centre FICAN West, Turku, Finland; ^3^ MediCity Research Laboratory, University of Turku, Turku, Finland; ^4^ InFLAMES Research Flagship Center, University of Turku, Turku, Finland; ^5^ Turku Center for Disease Modeling, University of Turku, Turku, Finland; ^6^ Turku Bioscience Centre, University of Turku and Åbo Akademi University, Turku, Finland; ^7^ Department of Oncology, Tampere University Hospital, Tays Cancer Center, Tampere, Finland; ^8^ Department of Oncology and Radiation Therapy, Oulu University Hospital, Oulu, Finland; ^9^ Cancer Research and Translational Medicine Research Unit, University of Oulu, Oulu, Finland

**Keywords:** CD73, TNBC, zoledronate, tumor growth, tumor-infiltrating lymphocytes

## Abstract

**Introduction:**

Bisphosphonates (BPs) are bone-protecting osteoclast inhibitors, typically used in the treatment of osteoporosis and skeletal complications of malignancies. When given in the adjuvant setting, these drugs may also prevent relapses and prolong overall survival in early breast cancer (EBC), specifically among postmenopausal patients. Because of these findings, adjuvant nitrogen-containing BPs (N-BPs), such as zoledronate (ZOL), are now the standard of care for high-risk EBC patients, but there are no benefit-associated biomarkers, and the efficacy remains low. BPs have been demonstrated to possess anti-tumor activities, but the mechanisms by which they provide the beneficial effects in EBC are not known.

**Methods:**

We used stably transfected 4T1 breast cancer cells together with suppression of CD73 (sh-CD73) or control cells (sh-NT). We compared ZOL effects on tumor growth and infiltrating lymphocytes (TILs) into tumors and lung metastases using two mouse models. B cell depletion was performed using anti-CD20 antibody.

**Results:**

Sh-CD73 4T1 cells were significantly more sensitive to the growth inhibitory effects of n-BPs *in vitro*. However, while ZOL-induced growth inhibition was similar between the tumor groups *in vivo*, ZOL enhanced B and T lymphocyte infiltration into the orthotopic tumors with down-regulated CD73. A similar trend was detected in lung metastases. ZOL-induced tumor growth inhibition was found to be augmented with B cell depletion in sh-NT tumors, but not in sh-CD73 tumors. As an internal control, ZOL effects on bone were similar in mice bearing both tumor groups.

**Discussion:**

Taken together, these results indicate that ZOL modifies TILs in breast cancer, both in primary tumors and metastases. Our results further demonstrate that B cells may counteract the growth inhibitory effects of ZOL. However, all ZOL-induced TIL effects may be influenced by immunomodulatory characteristics of the tumor.

## Introduction

Bisphosphonates (BPs) inhibit osteoclast-mediated bone resorption and thereby, effectively prevent osteoporotic bone fractures in osteoporosis and skeletal complications in bone metastasis (1). These drugs have also well documented anticancer effects (2–4). For example, BPs induce cancer cell apoptosis and prevent tumor growth *in vivo* (5–7). It has also been demonstrated in a large meta-analysis that BPs, when given in the adjuvant setting, provide survival advantage to a small fraction of breast cancer patients (8). This effect was detected with both pyrophosphate-like (p-BP) and N-BPs and was mostly due to prevention of bone metastasis. The protective effect was specifically detected among postmenopausal women (9, 10). Despite their well-characterized effects on the mevalonate pathway or on the production of ATP-like metabolites in cells (11), it remains unknown how adjuvant BPs prevent the outgrowth of microscopic disease into clinically detectable metastases (8). Furthermore, adjuvant BPs provide a survival effect for only 3% of breast cancer patients. Thus, the patient numbers needed to treat for one person to gain improved survival remains high. Although some prognostic biomarkers have been proposed, they are not yet in clinical use (12).

CD73 is a glycosylphosphatidylinositol-anchored membrane protein, which hydrolases AMP to adenosine and inorganic phosphate. A high CD73 expression has been reported in various cancer types, such as triple-negative breast cancer (TNBC) (13), pancreatic (14), gastric (15) cancer cells, renal cell carcinoma (16), esophageal squamous cell carcinoma (17) or lung adenocarcinoma (18). We and others showed that CD73 facilitates breast tumor growth in a pre-clinical model (19, 20). Low tumor CD73 expression is also associated with improved survival in TNBC. Moreover, a recent study demonstrated that low tumor CD73 expression levels were associated with higher pathologic complete response rates in TNBC patients receiving neo-adjuvant chemotherapy. These findings have raised interest in CD73 as a molecular target and currently, there are several active clinical trials investigating the effect of CD73 inhibition in cancer (21, 22).

Both BPs and CD73 regulate immune responses. Especially the newer, N-BPs are proinflammatory. They increase cytokine release and expand gamma-delta T cell populations, which are associated with cytotoxic effects against cancer cells (23). Furthermore, regulatory T cell expansion was suppressed in cell cultures using conditioned media from zoledronate pre-treated TNBC cells (24). CD73 and adenosine, on the other hand, have an immunosuppressive role in cancer progression (25). For example, blockage of adenosine production activated immune cells within the tumor microenvironment, along with sensitizing cancer cells to anti-cancer therapy (26). The correlation between elevated CD73 expression and unfavorable outcomes in TNBC may also be attributed to the impact on the immune system. Adenosine assists cancer cells in evading the immune system’s attempts to target and eradicate them. As a result, TNBC tumors with high CD73 expression might be shielded from the body’s inherent immune responses against tumors, ultimately resulting in a poorer prognosis for patients (21). The role of tumor infiltrating lymphocytes (TILs) is gaining importance in the pathophysiology and treatment of breast cancer (27). The aim of this study was to investigate whether zoledronate affects TILs. We also investigated whether CD73-dependent, tumor immunosuppressive characteristics affect N-BP responses in TNBC tumors.

## Materials and methods

### Cells

Human MDA-MB-231 and mouse 4T1, representing TNBC cells and human T47-D cells, representing luminal A type breast cancer cells (all from ATCC, Manassas, VA, USA) were cultured as previously described (28). CD73 was downregulated in the 4T1 cells through stable small hairpin RNA (shRNA) transduction, using mouse-specific lentiviral particles, according to manufacturer’s recommendations (Mission lentiviral transduction particles, Sigma-Aldrich) as described previously (20).

### RNA sequencing

RNA-Seq (RNA sequencing) service was performed by LC Sciences (Houston, Texas) to analyze 4T1 sh-NT and 4T1 sh-CD73 cells. Poly(A) RNA sequencing library was prepared following Illumina’s TruSeq-stranded-mRNA sample preparation protocol. RNA integrity was checked with Agilent Technologies 2100 Bioanalyzer. Poly(A) tail-containing mRNAs were purified using oligo-(dT) magnetic beads with two rounds of purification. Cutadapt (29) and perl scripts in house were used to remove the reads that contained adaptor contamination, low quality bases and undetermined bases. The sequence quality was verified using FastQC (http://www.bioinformatics.babraham.ac.uk/projects/fastqc/). HISAT2  (30) was used to map reads to the genome of ftp://ftp.ensembl.org/pub/release-101/fasta/mus_musculus/dna/. StringTie  (31) was used to perform expression level for mRNAs by calculating FPKM. mRNAs differential expression analysis was performed by R package DESeq2  (32) between two different groups (and by R package edgeR  (33) between two samples). The mRNAs with the parameter of false discovery rate (FDR) below 0.05 and absolute fold change ≥ 2 were considered differentially expressed mRNAs. Database links presented in **Supplementary Table 1**.

### Cell viability assay

Cancer cells were seeded in 96-well plates (2×10^3^ cells/well) and left to attach overnight. IC_50_ of N-BPs (zoledronate = ZOL, alendronate = ALN, pamidronate = PAM) for sh-NT and sh-CD73 cells was measured using 6 technical replicates after 72 h of treatment. N-BP concentrations varied from 1 µM to 500 µM followed by 50% serial dilutions to lower doses. The IC50 values were obtained by non-linear regression analysis using GraphPad Prism version 7.0 (GraphPad Software Inc, San Diego, CA, USA). Obtained IC_50_ values for individual cell lines were used throughout the study. Additionally, cell viability was measured upon 100 µM Adenosine 5’-(α,β-methylene) diphosphate (APCP, Merck Life Science OY, Finland) treatment after 72h. Cell viability was measured by WST-8 assay (Dojindo, Biotop Oy, Denmark). The level of WST-formazan was quantified using a microplate Tecan ULTRA Reader (Tecan AG, Austria) at 450 nm.

### CD73 analyses

For quantitative PCR, cells at the density of 10^4^ cells were cultured with IC_50_ N-BP concentrations in 6-well plates (Corning, USA) for 72 h. Quantitative PCR was performed using SYBR Green qPCR kit (Bio-Rad) as previously described by us (20). For analysis of CD73 activity, cells were seeded onto 96-well flat bottom clear plates at a density of 1×10^4^ cells/well and let to attach overnight. Cells were treated for 72h with N-BPs prior to addition of [^3^H] AMP substrate. CD73 activity was determined by thin-layer chromatographic (TLC) analysis as was described before (34).

### IncuCyte measurements

Cells were seeded onto 96-well plates (2×10^3^ cells/well) and allowed to attach overnight. For proliferation studies, cell growth after N-BPs treatment was assessed for 72 h, to allow cells to reach confluency. For caspase3/7 measurement, ZOL and caspase 3/7 (4704, Sartorius) reporter red dye (ratio 1:8) were added for 72 h. Apoptotic cells showed cleaved caspase 3/7 staining in the nucleus, which was shown by the appearance of red fluorescence emission in IncuCyte S3. Cell density and the number of caspase3/7-positive cells were analyzed using IncuCyte S3 with IncuCyte 2020A software (Sartorius).

### Flow cytometry analysis

Cell cycle assay was performed with Click-iT™ EdU Pacific Blue™ (ThermoFisher Scientific). Apoptosis assay was performed with Annexin V-FITC Apoptosis Staining/Detection Kit (ab14085, Abcam). Cells were seeded onto 6-well plates (3×10^4^ cells/well) and allowed to attach overnight. Next, cells were treated with N-BPs and incubated for 72 h. Cell pellets were collected and stained according to the kit protocols. Samples were analyzed using BD LSRFortessa flow cytometer (BD Biosciences). The data was analyzed with Flowing Software 2.5.1 (Perttu Terho, Turku Bioscience Centre, Turku, Finland).

### Western blotting

Cells were cultured in complete culture medium and harvested after 72 h of N-BPs treatment in RIPA buffer (Thermo Fisher Scientific). Protein amounts were measured using bicinchoninic acid (BCA) protein assay (Thermo Fisher Scientific). The membranes were incubated with 5’-Nucleotidase/CD73, Caspase-3, p27 and α-tubulin primary antibodies overnight at 4°C (**Supplementary Table 2**). Secondary detection was performed with anti-rabbit 800CW and anti-mouse 680CW antibodies (1:2000, IRDye, LI-COR). The emitted fluorescence was detected with Li-Cor Odyssey CLx imaging system.

### *In vivo* experiments

Four-week-old female Balb/c mice (Balb/cOlaHsd) were obtained from Envigo (Netherlands). Animals were maintained under controlled pathogen-free environmental conditions with a 12h light/dark cycle. Mice were inoculated with sh-NT and sh-CD73 4T1cells (2×10^4^ cells in 100 μl PBS per mouse) orthotopically into 4^th^ mammary fat pads (n = 10/group) and followed for 31 days. For the metastasis models, the mice were inoculated intravenously with sh-NT and sh-CD73 4T1 cells (5×10^4^ cells in 100 μl PBS per mouse) into tail vein (n = 6/group) and followed for 20 days. In the B cell depletion model, 100 µM/animal Ultra-leaf purified anti-mouse CD20 (BioLegend, 152104) and control IgG antibody (BioLegend, 400671) were injected intravenously in the tail vain, once cells were inoculated and followed for 34 days. Animals were treated intraperitoneally each 4^th^ day with the dose of 6 µg ZOL/animal. Body weights and tumor dimensions (35) were measured once a week. The animals were sacrificed when weight loss was ≥ 10% (data not shown).

### Analysis of the B cell depletion efficiency

After sacrifice, spleen and lung samples were mashed through 70 µm strainer (22363548, Fischer scientific) to a new well. The strainer was washed with MAC buffer (2mM EDTA, 0,5% BSA, 1 x PBS). Isolated cells were incubated for 5 min RT with red blood lysis buffer (420301, Biolegend). The reaction was stopped with 1 x PBS. 1 x 10^6^ cells were spined down (5 min, 500 x G) and resuspend in 2% BSA, 1 x PBS, 2 µL TruStain FcX (101320, Biolegend). Total cellular fraction isolated from lungs were analyzed from the presence of lymphocytes. Specifically, the isolated cells were incubated with anti-CD8 and anti-CD19 antibodies according to manufacturer’s recommendations (**Table S2**). Isolated spleen cells were incubated with conjugated CD19/CD3 antibodies (**Table S2**) for 1 h at 4°C in dark. Blood was drawn with intracardiac punctures into anti-coagulated K2E tubes (BD Microtainer, 1307939). Whole blood was stained with conjugated anti-CD19 antibody for 1 h at 4°C in dark (**Table S2**). All samples were washed with cell staining buffer (BioLegend, 420201) and centrifugated for 5 min at 500 g. Cell pellet was resuspended in 500 µL of cell staining buffer. The presence of CD19-positive cells was analyzed using flow cytometry (BD LSRFortessa, BD Biosciences). The data was analyzed with Flowing Software 2.5.1 (Turku, Finland).

### Histology and tissue staining

Dissected tumors and lungs were fixed with 10% paraformaldehyde for 24 h, after which they were processed into paraffin blocks and cut tissue sections with standard methods (20). Dissected lungs were stained with hematoxylin and eosin staining. For IHC staining, dissected tumors were stained immunohistochemically to analyze cleaved caspase-3 (cCas-3), phospho-histone H3 (pHH3), CD34, CD45R/B220 and CD4 cells (**Table S2**). Slides were scanned using Pannoramic 250 slide scanner (3DHISTECH Ltd, Hungary). For immunofluorescent staining, dissected tumors were stained with anti-CD8 AlexaFluor 488 and Ki-67 antibodies (**Table S2**). Secondary anti-rabbit AlexaFluor 488 antibody was applied for 1h at RT. DAPI was used as a nuclear counterstain. Slides were scanned using Pannoramic Midi fluorescence slide scanner (3DHISTECH Ltd, Hungary). Acquired digital slides were analyzed with QuPath-0.2.0 software (36). All stainings were evaluated blindly. QuPath scripts used for image analysis are presented in **Table S3.**

### Bone analyses

For bone histology, tibiae were dissected and prepared into paraffin-blocks and cut sections, as previously described (37). Osteoclasts were stained for tartrate-resistant acid phosphatase (TRAP) (Merck, Germany). The number of osteoclasts were counted per area in the trabecular bone manually using Fiji-ImageJ (1.52p) software. Quantitative analysis of femurs was performed using a Skyscan 1272 X-ray computer tomography scanner (Bruker, Kontich, Belgium). Morphometric parameters including tissue volume (TV, mm^3^), bone volume (BV, mm^3^) and bone volume/tissue volume (%) were analyzed by CTan version 1.9.32 software from Skyscan. The parameters applied for scanning were the following: x 26.31 magnification, X-ray tube voltage 61 kV, tube current 148 μA, X-ray filtration with 0.25 mm aluminum filter. Trabecular bone morphometric region of interest was defined at metaphysis of the femur starting 11 layers (122μm) below an anatomic marker, showing lower surface of the growth plate and extending 50 layers (557μm).

### Statistical analysis

Results are showed as the mean ± SD of independent experiments with parallels. All analyses were performed using GraphPad Prism version 7.0 (GraphPad Software Inc, San Diego, CA, USA). Data were analyzed for statistical significance using Mann-Whitney t-test, one-way and two-way analysis of variance (ANOVA). Differences for which *P* was <0.05 are reported as statistically significant. Original dataset is available in a publicly accessible repository. This data can be found here:

### Ethical approval

All procedures involving animal studies were cared for in accordance with the Project Authorization Board of Finland (license No ESAVI/7015/2020) in accordance with the 2010/EU/63 EU Directive on the protection of animals used for scientific purposes and the ARRIVE guidelines (38).

## Results

### CD73 gene involvement in cell cycle and inflammatory pathways in 4T1 cancer cells

**Figure 1:**
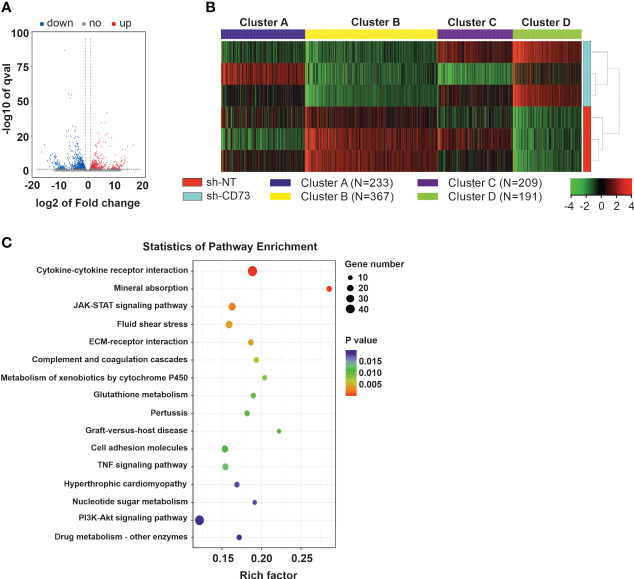
Gene expression in 4T1 sh-CD73 vs sh-NT cells. **(A)** Volcano map of the differential gene expression. **(B)** Differentially expressed gene clusters between sh-CD73 and sh-NT cells, using three replicates. The clusters were defined using the kmeans algorithm, using iDEP tool available online. **(C)** The KEGG diagram was made according to the gene pathway enrichment. The mRNAs with the parameter of false discovery rate (FDR) below 0.05 and absolute fold change ≥ 2 were considered differentially expressed mRNAs. The gene expression signature of 4T1 sh-NT and sh-CD73 cells were determined by RNA sequence (LC Sciences, Houston, Texas).

We have previously demonstrated that suppression of CD73 expression affects migration and viability of TNBC cells (20). To further characterize CD73 shRNA-induced changes in these cells, sh-NT and sh-CD73 cells were analyzed with RNA-seq. The analysis revealed 551 upregulated (log2 (fc) > 1, p < 0.05) and 886 downregulated (log2 (fc) < 1, p < 0.05) genes in sh-CD73 cells as compared with sh-NT cells (**Figure 1A** and **Supplementary File 2**). We then used k-means clustering to divide the top 1000 most variable genes from RNA-seq FPKM (fragments per kilobase of exon per million mapped fragments) data into clusters *via* iDEP tool (39). We identified 4 clusters based on GO Biological Process database. Three clusters were involved in inflammation and immune responses and one cluster in cell division and replication (**Figure 1B** and **Figure S1**). Additionally, we applied KEGG enrichment analysis on the most engaged pathways changed in sh-CD73 versus sh-NT cells (**Figure 1C**). The genes that passed the threshold level (log2 (fc) > 1.5 or log2 (fc) < – 1.5, p > 0.05) in the pathways were associated with apoptosis, cell cycle and cytokine activity and are presented in **Supplementary Table 4**.

### Suppression of CD73 expression sensitizes TNBC cells to bisphosphonates *in vitro*.

To begin our studies, we first wanted to define whether CD73 expression in TNBC alters direct cellular response to N-BPs. We treated 4T1 sh-NT and sh-CD73 cells with ZOL, ALN and PAM, and determined the IC_50_ doses with cell viability assays (**Table S5**). Sh-CD73 cells were significantly more sensitive than sh-NT cells to ZOL and ALN IC_50_ doses after 48h, and to all selected N-BPs after 72h (**Figures S2A, B**). Thus, we selected ZOL and ALN for further experiments. N-BPs did not directly affect CD73 catalytic activity, mRNA or protein expression level (**Figures S2C–E**). We also tested the combined effects of APCP, a specific CD73 activity inhibitor and ZOL in parental cells. APCP did not augment ZOL effects on cell viability of any breast cancer cell lines (**Figures S3A–C**). Thus, our results suggest that suppression of CD73 expression, but not enzymatic activity sensitizes cells to N-BPs *in vitro*.

### Suppression of CD73 expression delayed cell proliferation and induced apoptosis upon bisphosphonates

**Figure 2:**
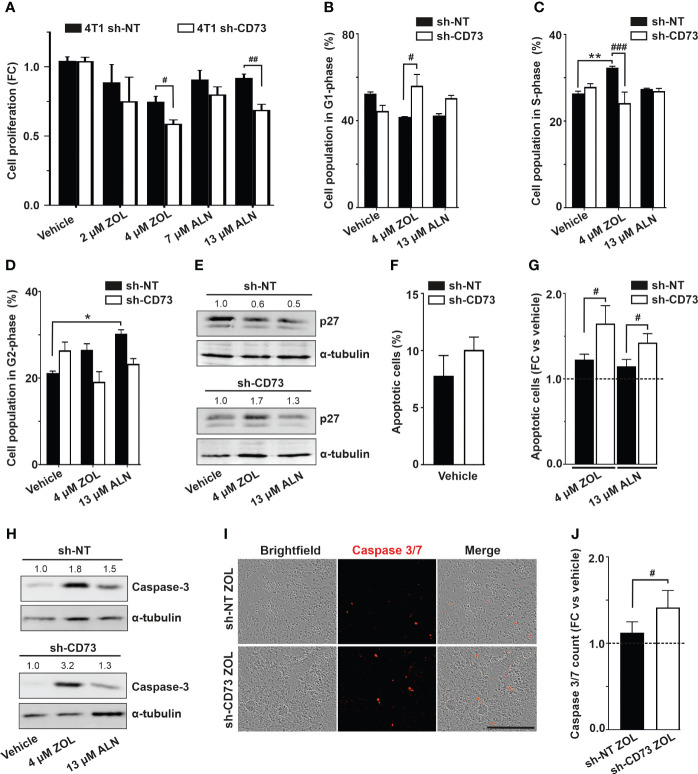
Suppression of CD73 causes cell cycle arrest and increases apoptosis upon N-BP treatment. **(A)** Cell proliferation of sh-NT and sh-CD73 4T1 cells upon N-BPs treatment for 72 h. Cell proliferation was assessed with confluence analysis using IncuCyte 2018B software (Essen Bioscience). The percentage of cells **(B)** in G1 phase, **(C)** S phase, **(D)** G2 phase of cell cycle upon N-BPs treatment for 72 h. **(E)** Representative dot plots of p27 protein expression upon N-BP treatment. **(F)** The percentage of apoptotic cells in vehicle and **(G)** N-BPs treated sh-NT and sh-CD73 groups. **(H)** Representative dot plots of caspase-3 protein expression upon N-BP treatment. **(I)** Representative images of caspase 3/7 staining. The images were generated by IncuCyte 2018B software (Essen Bioscience). **(J)** The number of caspase 3/7 positive 4T1 cells upon zoledronate treatment for 72 h. The bars represent fold-change in number of caspase3/7 in sh-NT ZOL-treated vs. sh-CD73 ZOL-treated cells. The results are expressed as mean ± SD, n = 3. * P < 0.05, ** P < 0.01, comparing within the same group upon different treatment; # P < 0.05, ## P < 0.01, ### P < 0.001, comparing sh-CD73 treated cells vs. sh-NT cells treated cells.

Further experiments were conducted with IC_50_ concentrations at 72h. In line with decreased viability, ZOL and ALN caused a significant decrease of proliferation in sh-CD73 cells compared to sh-NT cells after 72h of treatment (**Figure 2A**). Significantly higher percentage of sh-CD73 cells than sh-NT cells were at G1-phase after ZOL-treatment (**Figure 2B**). Compared with vehicle, ZOL also significantly increased the percentage of sh-NT cells at S-phase. No such effect was seen in sh-CD73 cells (**Figure 2C**). Both ZOL and ALN increased sh-NT cell population in G2-phase compared to 7vehicle. In sh-CD73 cells, no such effect was seen (**Figure 2D**). Cyclin-dependent kinase inhibitor, p27 is a marker of cell cycle transition. We showed that sh-CD73 cells increased expression of p27 upon ZOL and ALN (**Figure 2E**). There was a trend of increased the percentage of apoptotic cells in vehicle treated sh-CD73 cells compared to sh-NT cells (**Figure 2F**). Both ZOL and ALN induced a significantly higher fold-increase in apoptosis in sh-CD73 cells in comparison to sh-NT cells after 72h treatment (**Figure 2G**), an effect which was not seen in in vehicle treated sh-CD73 cells. Apoptotic marker, caspase-3 was increased upon ZOL- and ALN-treatments. Furthermore, sh-CD73 cells demonstrated increased expression of caspase-3 upon ZOL-treatment in comparison to sh-NT cells (**Figure 2H**). In agreement with this, ZOL significantly increased the number of caspase 3/7 positive cells (**Figures 2I, J**) in sh-CD73 cells, as compared to sh-NT cells. Taken together, these results indicate that the increased sensitivity of sh-CD73 cells to the growth inhibitory effects of ZOL or ALN is due to changes in cycle arrest and increased apoptosis.

### ZOL increases tumor infiltrating lymphocytes in sh-CD73 tumors

**Figure 3:**
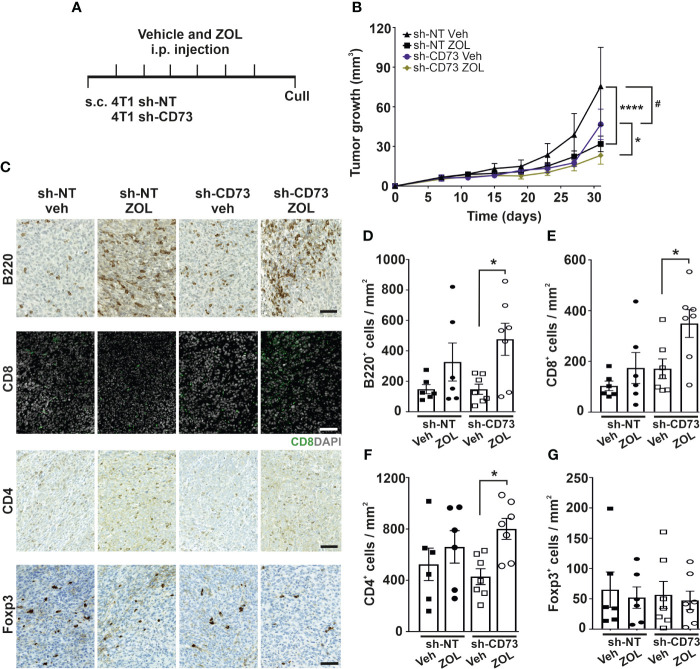
Zoledronate increases immune cell infiltration into CD73-suppressed tumors. **(A)** A schematic view of the in vivo experiment. Zoledronate was given at a dose of 6µg/animal for six times after tumors were formed. **(B)** sh-NT and sh-CD73 tumor growth demonstrated as a function of time. Tumor dimensions were measured with a caliper once a week. * P < 0.05, **** P < 0.0001, comparing within the same group upon different treatment; Data is expressed as mean ± SEM, by a two-tailed Student’s t – test. # P < 0.05, comparing sh-CD73 tumors vs. sh-NT tumors. **(C)** Representative images of B220, CD8, CD4 and Foxp3 stainings in sh-NT and sh-CD73 tumors. Scale bar 100 μm. Number of **(D)** B220-positive cells, **(E)** CD4-positive cells, **(F)** CD8-positive and **(G)** Foxp3-positive cells from 4T1 sh-NT and sh-CD73 tumors. Data is expressed as mean ± SEM, by a two-tailed Student’s t – test. * P < 0.05; sh-CD73 vs. sh-NT tumors.

As ZOL demonstrated the most effective growth inhibition of cells *in vitro*, we next compared effects of ZOL on sh-NT and sh-CD73 tumor growth *in vivo*, using an immune-competent, mammary fat pad mouse model of breast cancer (**Figure 3A**). As also seen previously (20), sh-CD73 cells formed significantly smaller tumors than sh-NT cells. Tumor growth was significantly suppressed in both ZOL-treated sh-NT (32%) and sh-CD73 (36%) groups compared to vehicle groups (**Figure 3B**). Unlike *in vitro*, ZOL-induced growth inhibition was similar in both tumor groups (**Figures S4A, B**). As an internal control for CD73 suppression throughout the experiment, significantly lower CD73 mRNA expression was maintained in the sh-CD73 tumors at sacrifice. In line with our *in vitro* results, ZOL did not influence CD73 mRNA expression in tumors either (**Figure S4C**). As an internal control for ZOL efficacy, we confirmed that ZOL significantly prevented bone resorption and decreased the number of osteoclasts in mice bearing either sh-NT or sh-CD73 tumors (**Figures S4D–G**).

There are several important characteristics, which implicate cancer progression, including proliferative status of tumor cells or their interaction with immune cells (40). Although, CD73 suppression in vehicle-treated tumors significantly decreased the number of pHH3^+^ cells (mitotic marker) in comparison to vehicle-treated sh-NT tumors, it did not affect the number of cleaved-Caspase3 (apoptotic marker) cells or CD34^+^ and CYR61^+^ (angiogenesis markers) cells in vehicle-treated tumors (**Figures S5B–E**). ZOL significantly increased the number of cleaved-Caspase3^+^ cells in sh-CD73 group compared to vehicle-treated sh-CD73 group. There was a trend of ZOL reducing pHH3+ cells in both groups (**Figures S5B–E**) and CD34^+^ cells in sh-CD73 tumors (**Figure S5C, D**). However, the treatment did not alter the number of CYR61^+^ cells (**Figure S5E**). Taken together, in agreement with the *in vitro* data, sh-CD73 tumors had more apoptotic cells after ZOL treatment than after vehicle-treatment. A similar trend was seen in sh-NT tumors, but none of the differences were statistically significant.

Immune cell infiltration into tumors can promote or suppress tumor progression. The interplay of immune cells in this context is, however, very complex. For example, B cell infiltration demonstrated anti-tumor activity, resulting in better OS of cancer patients, but in the presence of effector T-cells (41). There are previous reports on BP effects on TILs, especially on T-cell (42, 43), but whether N-BPs affect B cell infiltration into tumors, is not known. The number of TILs was similar between vehicle-treated sh-NT and sh-CD73 tumors (**Figures 3D–G**). Compared with vehicle-treated sh-CD73 tumors, ZOL significantly increased B220^+^ B cell, CD4^+^ and CD8^+^ T cell infiltration in sh-CD73 tumors. Only two tumors in the sh-NT group (n=6) showed increased numbers of B cells and CD8^+^ T cells upon ZOL (**Figures 3C–E**). ZOL treatment had no effect on FOXP3^+^ T helper cells in either group (**Figure 3F**). Thus, our results suggest that ZOL induces lymphocyte infiltration into primary tumors and that low CD73 expression in the tumor augments this effect.

### ZOL increases TIL infiltration into lung metastases

**Figure 4:**
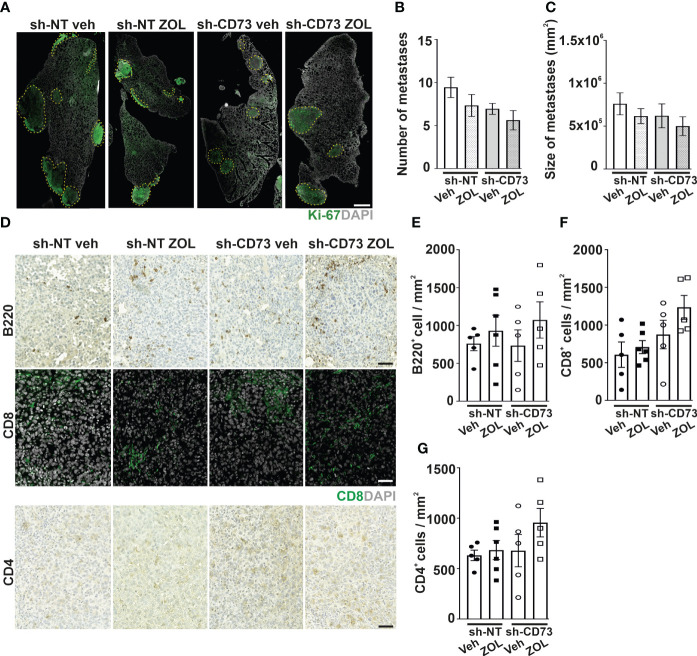
The effect of CD73 suppression on immune cell infiltration into lung metastases after zoledronate treatment. Cancer cells were injected intravenously into mouse tail veins (n = 6/group). Lung samples for staining were collected after 20 days. **(A)** Representative images of lung sections stained with antibody against Ki-67. Scale bar 500 μm. The number **(B)** and size **(C)** of lung metastases. **(D)** Representative images of B200, CD8 and CD4 immune cell stainings in lung metastases formed by sh-NT and sh-CD73 cells. Scale bar – 100 μm. Number of **(E)** B220-positive cells, **(F)** CD8-positive cells and **(G)** CD4-positive cells in lung metastases formed by sh-NT and sh-CD73 cells. Data is expressed as mean ± SEM.

We previously demonstrated that sh-CD73 cells formed significantly lower lung metastatic burden than sh-NT cells (20). ZOL had no obvious effects on the number and sizes of lung metastases in either group (**Figures S5F, G**). To investigate ZOL effects on TILs at lung metastases, we used an experimental lung metastasis model, which typically results in the formation of larger lung metastases without the engagement of primary tumors. With this model as well, there was a trend of fewer and smaller metastases formed by the sh-CD73 cells. ZOL, however, had no obvious effect on the number of metastases (**Figures 4A–C**). Similar to immune cell infiltration into mammary fat pad tumors, there was a trend towards ZOL-induced B220^+^ B cell, CD4^+^ and CD8^+^ T cells infiltration into lung metastases. This effect appeared to be slightly more pronounced in the sh-CD73 than in the sh-NT tumors, but none of these changes reached statistical significance (**Figures 4D–G**).

### B cell depletion augments ZOL effect on growth in CD73-expressing tumors

**Figure 5:**
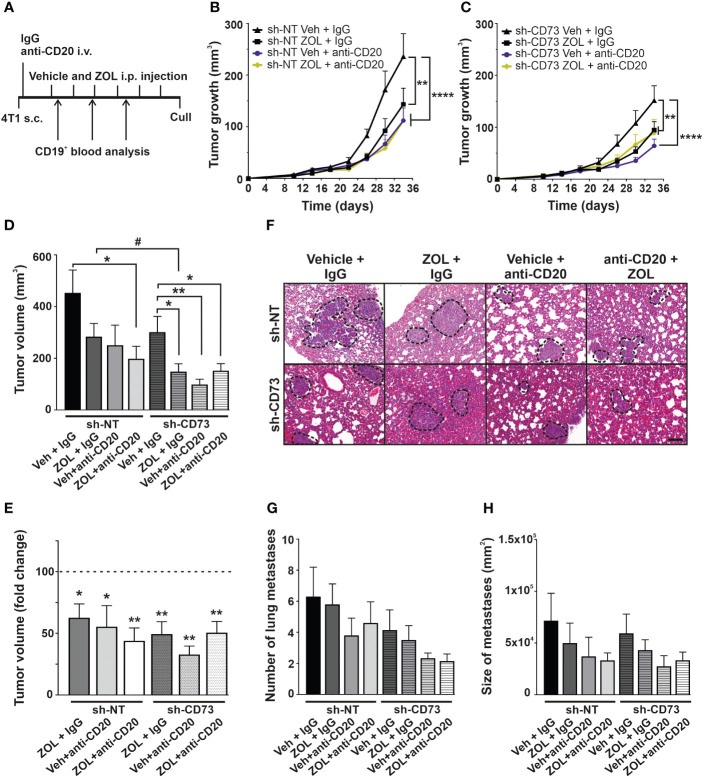
The effect of anti-CD20 and zoledronate treatment on tumor growth. **(A)** Schematic views of *in vivo* experiment. Animals were treatment with 100 µg/mouse anti-CD20 antibody after tumor cells were inoculated. Zoledronate was given at a dose of 6µg/animal for six times after tumors were formed. The number of circulating CD19-positive cells was analyzed throughout the experiment by Flow cytometry 3 times. **(B)** sh-NT and **(C)** sh-CD73 tumor growth upon treatment shown as a function of time. Tumor dimensions were measured with a caliper once a week. **(D)** Tumor volume and **(E)** fold-change of tumor volume at the sacrifice. **(F)** Representative images of H&E staining of lungs. Scale bar 200 μm. The number **(G)** and size **(H)** of lung metastases from 4T1 sh-NT and sh-CD73 cells. Data is expressed as mean ± SEM, by one-way ANOVA with a Sidak post-test. * P < 0.05, ** P < 0.01 and **** P < 0.0001, comparing within the same group upon different treatment. # P < 0.05, comparing sh-CD73 treated tumors vs. sh-NT cells treated tumors.

TNBC tumors have been suggested to have higher levels of B cell infiltration than other breast cancer subtypes, but their role in the disease pathophysiology is unclear (3, 9–21, 23–41). Therefore, we further explored the role of B-cells in our model in general, and also whether they contribute to ZOL effects in tumors (**Figure 5A**). We first determined an effective dose of B cell-depleting anti-CD20 antibody, by assaying its effect on circulating B cells using CD19 as a marker. A single dose of anti-CD20 IgG (100µM/animal) efficiently reduced the absolute number of CD19^+^ lymphocytes in spleens, compared to control IgG group. Anti-CD20 treatment also slightly increased the absolute number of CD3^+^ lymphocytes in spleen compared to control IgG group (**Figure S6**). This dose was used in further experiments. In the mouse orthotopic tumor model, both ZOL or anti-CD20 treatment alone significantly reduced tumor growth in sh-NT and sh-CD73 tumors, as compared with corresponding controls. The effect of anti-CD20 appeared to be slightly stronger in sh-CD73 tumors. No significant synergistic effects of ZOL and anti-CD20 were seen in sh-NT tumors. (**Figures 5B, C**). Notably however, whereas in sh-NT tumors there was a trend of anti-CD20 antibody further augmenting ZOL-induced growth inhibition, no such effect was detected in the sh-CD73 tumors (**Figures 5B, C**). Additionally, the post-mortem analysis suggested that ZOL significantly reduced tumor size in sh-CD73 group in comparison to sh-NT group. ZOL + anti-CD20 treatment could be more efficient that the individual treatments in sh-NT tumors, while in sh-CD73 tumors both ZOL and anti-CD20 seemed to have similar effect without further synergy (**Figures 5D, E**). The analysis of lung metastases showed the fewest and smallest metastases in the mice treated with anti-CD20, both with tumor cells expressing normal or reduced levels of CD73 and no clear synergy between ZOL and anti-CD20 was detected (**Figures 5F–H**). Taken together, the B cell depletion caused at least similar if not stronger growth inhibitory effects than ZOL in both tumor types and our data additionally suggested that sh-NT tumor could show some level of synergistic response, which was absent in sh-CD73 tumors.

### The effect of B cell depletion upon ZOL on immune cell infiltration into tumors

**Figure 6:**
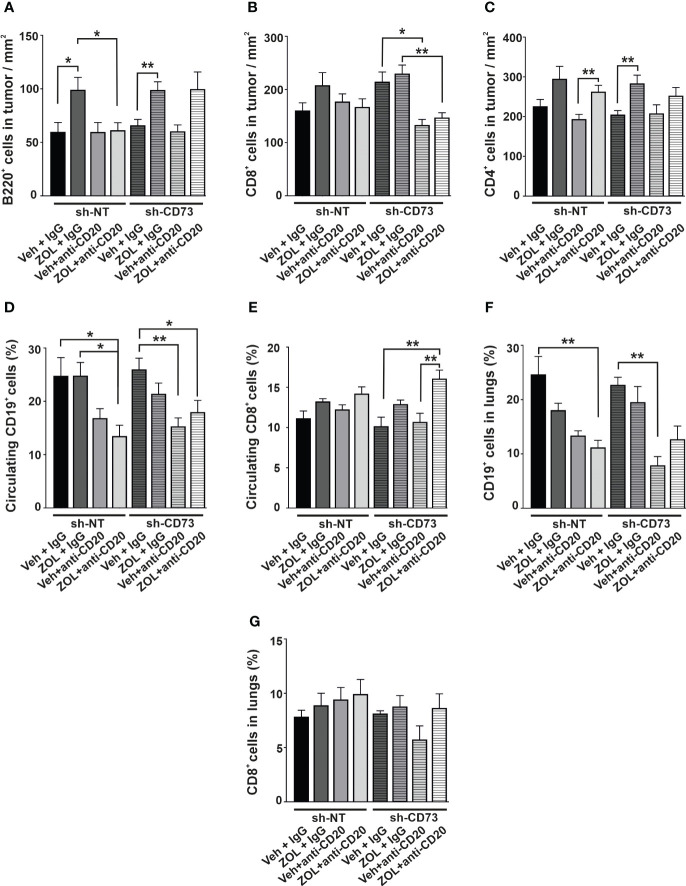
The effect of anti-CD20 treatment and zoledronate on circulating and tumor-infiltrating immune cells. The number of **(A)** B220-positive cells, **(B)** CD8-positive cells and **(C)** CD4-positive cells from 4T1 sh-NT and sh-CD73 tumors. The percentage of circulating **(D)** CD19-positive and **(E)** CD8-positive cells from 4T1 sh-NT and sh-CD73 tumor-bearing mice. The percentage of **(F)** CD19-positive and **(G)** CD8-positive cells in lungs from 4T1 sh-NT and sh-CD73 tumor-bearing mice. Data is expressed as mean ± SEM, by one-way ANOVA with a Sidak post-test. * P < 0.05, ** P < 0.01, comparing within the same group upon different treatment.

In the orthotopic tumor model, ZOL significantly increased B220^+^ B cell infiltration into both sh-NT and sh-CD73 tumors, in comparison to corresponding vehicle + IgG treatment (**Figure 6A**). Anti-CD20 treatment did not alter the baseline number of B220^+^ B cells in sh-NT and sh-CD73 tumors in comparison to vehicle-treated groups. However, anti-CD20 treatment removed ZOL-induced B220^+^ B cell infiltration in sh-NT tumors, but not in sh-CD73 tumors (**Figure 6A**). None of the treatments significantly affected CD8^+^ T cell infiltration in the sh-NT group. However, the number of CD8^+^ TILs was significantly suppressed by anti-CD20, with or without ZOL in sh-CD73 group (**Figure 6B**). ZOL seemed to increase CD4^+^ T-cell infiltration in both groups, showing significant difference in the sh-CD73 tumors. The combination of anti-CD20 + ZOL significantly increased CD4^+^ T-cell infiltration in comparison to anti-CD20 alone in the sh-NT group. This effect was not significantly affected by anti-CD20 in the sh-CD73 group (**Figure 6C**).

**Figure 7:**
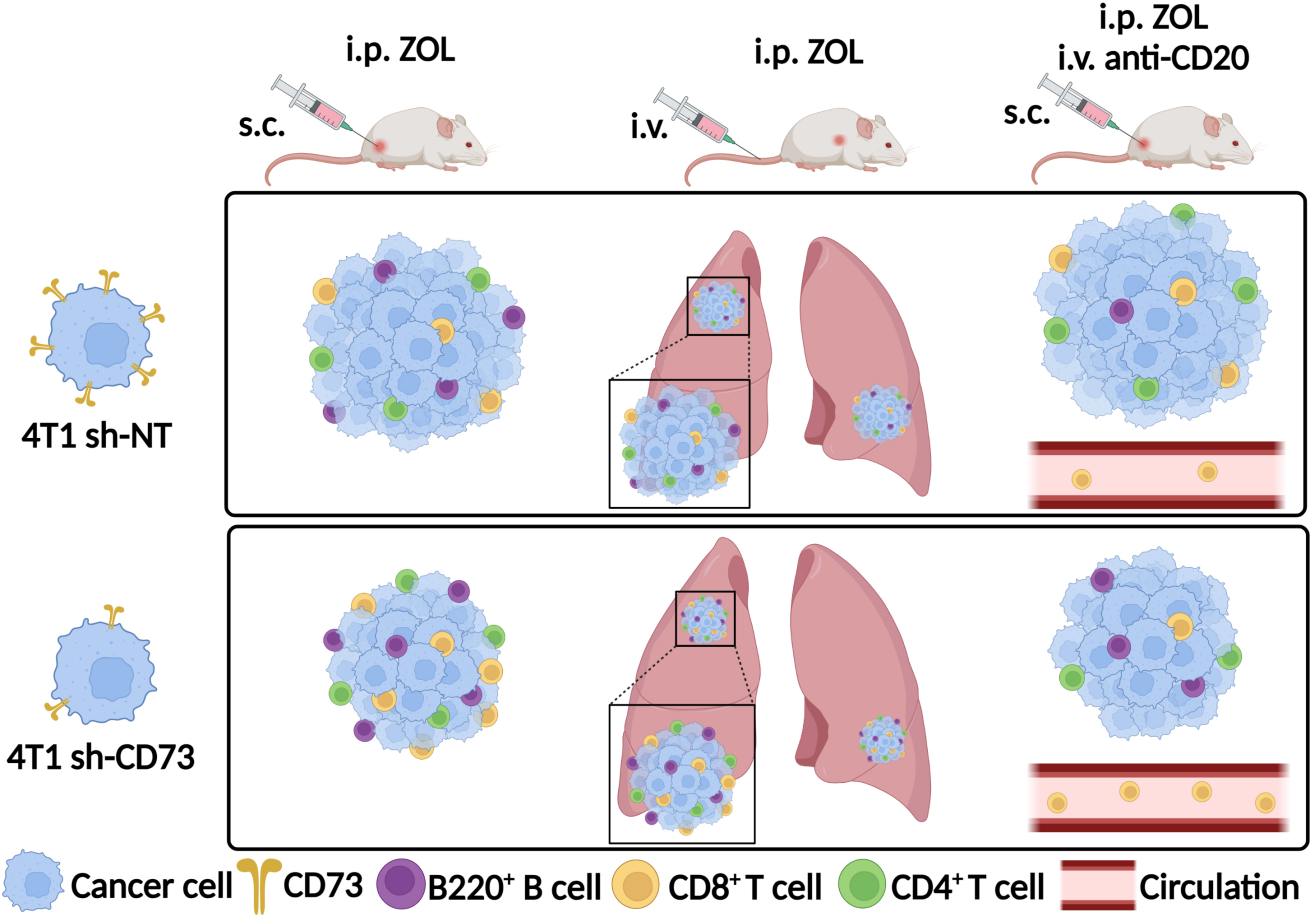
Zoledronate promotes B220+ B, CD8+ and CD4+ T cell infiltration into tumors or lung metastases with low CD73 expression. Depletion of B cells with anti-CD20 antibody led to reduced CD8+ T cell infiltration into tumors with low CD73 expression upon zoledronate-treatment. Zoledronate increased the number of CD8+ T cells in circulation when B cells were depleted in sh-CD73 tumor-bearing mice. ZOL, zoledronate; s.c., subcutaneously; i.v., intravenously; i.p., intraperitoneally; 4T1 sh-NT, cells were transfected with non-targeting particles; 4T1 sh-CD73, cells were transfected with a stable small hairpin RNA transduction, using mouse-specific lentiviral particles. Created with BioRender.com.

ZOL alone had no significant effect on the percentage of circulating CD19^+^ cells in either mouse group. In mice bearing sh-NT tumors, anti-CD20 antibody decreased circulating CD19^+^ cells, and this effect reached significance only upon anti-CD20 + ZOL. In mice bearing sh-CD73 tumors anti-CD20 and anti-CD20 + ZOL significantly decreased circulating CD19^+^ cells (**Figure 6D**). Although neither treatment alone had a significant effect, the combination of anti-CD20 + ZOL significantly increased the number of circulating CD8^+^ cells in comparison to corresponding vehicle in mice bearing sh-NT tumors. The effects were similar, but more pronounced in mice bearing sh-CD73 tumors (**Figure 6E**). We also investigated TILs in lung tissues with metastases, as our experimental metastases model demonstrated that lungs were a metastatic niche for 4T1 cells. The distribution of CD19+ cells in the total number of cells isolated from lungs mimicked those detected in blood (**Figure 6F**). Although, the changes were not significant, anti-CD20 + ZOL treatment resulted in highest CD8^+^ infiltrating cells in the lungs of mice bearing sh-NT tumors. Anti-CD20 decreased CD8 + TILs in the lungs of mice bearing sh-CD73 tumors, but adding ZOL attenuated this effect (**Figure 6G**). Taken together, our data shows that anti-CD20 treatment alone significantly inhibits tumor growth in both sh-NT and sh-CD73 tumors, suggesting that B-cells regulate TNBC growth, regardless of tumor CD73 expression status. ZOL induces B cell infiltration into tumors, and this may counteract the growth inhibitory effects of this drug. However, tumor CD73 expression may interfere with this effect, making tumors less permissive for CD8 cells. The main immunological findings of this study are depicted in **Figure 7**.

## Discussion

Adjuvant bisphosphonates increase the survival rate of postmenopausal women across different subtypes of breast cancer (44). The mechanism how this survival advantage is reached, is unclear and there are no predictive biomarkers for patient selection either. Especially N-BPs are pro-inflammatory and have been demonstrated to increase circulating immune cells both in pre-clinical and clinical studies (23). Less is known about their ability to affect tumor immunity. We studied here the effects of ZOL on tumor infiltrating lymphocytes. We further investigated whether immune system modulating tumor characteristics, namely CD73 expression, affects the growth inhibitory and inflammatory responses to N-BPs. Modulation of CD73 expression in the tumors was chosen, due to its prognostic significance and because it is a promising immunotherapeutic target especially in TNBC (21, 45, 46).

Our results demonstrate that CD73 suppression sensitizes 4T1 breast cancer cells to the growth inhibitory effects of N-BPs *in vitro*. These drugs, especially the most potent and clinically most frequently used N-BP, ZOL, paused the sh-CD73 4T1 cells at the G1-phase, delayed proliferation and increased apoptotic rate. These differences were not, however, reflected *in vivo*, as the tumor growth inhibitory responses to ZOL were similar regardless of the tumor CD73 expression rate.

N-BPs have well characterized pro-inflammatory effects. They have been shown to inhibit the migration of macrophages (47) and promote their polarization (48, 49), activate γδ T-cells, and increase the production of inflammatory mediators (43). It was also shown also that ZOL reduced infiltration of the immunosuppressive regulatory T cells (42). Here, we take these findings further and demonstrate, that ZOL also induces also B cell accumulation into the primary tumors and also into lung metastases. Our results also suggest that anti-CD20 antibody may weaken the growth inhibitory effects of ZOL in tumors with low CD73 expression. This suggests that under certain conditions, the infiltration of B-cells may oppose the growth inhibitory effects of this N-BP. This effect was partially regulated by tumor CD73 expression, suggesting that immunoregulatory characteristics of the tumor could modify the B-cell responses induced by ZOL. Anti-CD20 treatment, when given alone significantly inhibited tumor growth regardless of tumor CD73 expression, suggesting that eradication of B cells is a beneficial anti-tumor treatment approach in general. CD73 suppression made tumors less permissive for CD8 T cells upon ZOL treatment when B cells were depleted, without reducing CD8 T cells number in circulation or lungs. A previous study showed that inhibition of CD73 enzymatic activity did not influence CD8 T cells infiltration to tumors in mice with B cell depletion (50). Tumor sizes were assessed with caliper measurement in our experiments. This approach measures total tumors, including tumor infiltrating non-malignant cells, such as TILs. Thus, a possible explanation for the lack of difference in sensitivity to N-BPs between sh-NT and sh-CD73 cells, which was observed *in vitro*, but not *in vivo*, may partially be explained by differences in the immune cell responses that we detected. Furthermore, CD20 antibody could target not only CD20-positive B cells, but CD20-positive CD8 or CD4 T cells. This T cells subset showed the same activity as CD20-negative T cells (51), depletion of which could improve treatment for patients with multiple sclerosis (52). Given that cytotoxic activity of CD8 T cells against cancer cells, these CD20-posivite T cells could play role in cancer suppression as well, which requires further studies. Our finding is in agreement with previous publications demonstrating that anti-CD20 treatment decreases tumor growth in various cancer models (42, 43) and ZOL effects on B cell (53, 54).

There are several implications of our finding. First, immune surveillance plays a critical role in tumor progression (55). Thus, it could be, that it is the inflammatory, TIL promoting effects of adjuvant N-BPs that prevent the outgrowth of microscopic disease into macroscopic metastasis in post-menopausal women. This hypothesis is supported also by the fact that the benefit is seen in post-menopausal women, who are not immunosuppressed by estrogen, like younger women (56). Second, breast cancers are considered immunologically “cold tumors”, due to modest inflammatory infiltration (57). Converting immunologically cold tumors into hot is a major topic in immuno-oncology to improve responses to immunotherapy. Our results suggest that N-BPs should be further studied in this approach. Third, the role of B cells in tumor progression requires further analysis, since their role in cancer remains controversial (58, 59). B cells prevent tumor progression through releasing immunoglobulins and activation of T cells. However, the progression of tumor growth might also be promoted *via* B cell-induced immunosuppressive cytokines (60, 61). Further clinical studies are needed to examine N-BP treatment effects on TILs in breast and other cancers, and whether tumor baseline immunological features affect such outcomes.

## Data availability statement

The dataset presented in this study can be found in online repository. This data can be found here: https://www.ncbi.nlm.nih.gov/sra/, PRJNA950306.

## Ethics statement

The animal study was reviewed and approved by Project Authorization Board of Finland (license No ESAVI/7015/2020) in accordance with the 2010/EU/63 EU Directive.

## Author contributions

NP and KS designed the study. NP, AS, ST performed investigation. NP analyzed data. JM and JS contributed to experiment performance. PM and AJ contributed to experiment design. NP, JS and KS wrote the original draft. NP, AS, ST, JM, JS, PM, AJ and KS wrote and edited the manuscript. All authors contributed to the article and approved the submitted version.
