# Supplementary material for: CD73 regulates zoledronate-induced lymphocyte infiltration in triple-negative breast cancer tumors and lung metastases
Source: Front Immunol. 2023 Jul 18;14:1179022. doi: 10.3389/fimmu.2023.1179022 (PMC10390692; doi:10.3389/fimmu.2023.1179022)
Supplement: Supplementary file 1 [file DataSheet_1.docx]

**CD73 regulates zoledronate-induced lymphocyte infiltration in triple-negative breast cancer tumors and lung metastases**

**Nataliia Petruk^1^, Arafat Siddiqui^1,2^, Sina Tadayon^3^, Jorma Määttä^1,4^, Pieta K. Mattila^1,3,5,6^, Arja Jukkola^7^, Jouko Sandholm^6^, Katri S. Selander^8,9*^**

^1^Institute of Biomedicine, University of Turku, Turku, Finland

^2^Western Cancer Centre FICAN West, Turku, Finland
^3^MediCity Research Laboratory, University of Turku, Turku, Finland

^4^Turku Center for Disease Modeling, University of Turku, Turku, Finland

^5^InFLAMES Research Flagship Center, University of Turku

^6^Turku Bioscience Centre, University of Turku and Åbo Akademi University, Turku, Finland

^7^Department of Oncology, Tampere University Hospital, Tays Cancer Center, Tampere, Finland

^8^Department of Oncology and Radiation Therapy, Oulu University Hospital, Oulu, Finland

^9^Cancer Research and Translational Medicine Research Unit, University of Oulu, Oulu, Finland

*** Correspondence:**

Dr. Katri S. Selander, M.D, Ph.D.

Katri.Selander@ppshp.fi

**Keywords: CD73, TNBC, zoledronate, tumor growth, tumor-infiltrating lymphocytes**

| **Supplementary Table 1** | | |
| --- | --- | --- |
| **Database** | **Web links** | **Version/date** |
| Genome | ftp://ftp.ensembl.org/pub/release-101/fasta/mus_musculus/dna/ | v101 |
| Gene Orthology (GO) | http://geneontology.org | 2019.05 |
| KEGG | http://www.kegg.jp/ | 2019.05 |
|  | | |
| **Analysis item** | **Software** | **Version/date** |
| Quality control | FastQC | 0.10.1 |
| Adapter remove | Cutadapt | 1.10 |
| Mapping | HISAT | 2.0 |
| Transcripts assembly | StringTie | 1.3.4 |
| Differential expression analysis | DESeq2/edgeR | NA |
| GO and KEGG enrichment analysis | Perl scripts in house | NA |
| SNP/Indel analysis | samtools | 0.1.19 |
| SNP/Indel annotation | ANNOVAR | 2017.09 |
| Alternative splicing | rMATS | 4.1.1 |

| **Supplementary table 2**  List of antibodies | | | | |
| --- | --- | --- | --- | --- |
| Primary antibody | Western blotting | IHC/IF | Flow cytometry | Manufacturer |
| CD73 | 1:1000 | 1:200 |  | Novus, NBP2-158015 |
| p27 | 1:500 |  |  | Santa Cruz, sc-528 |
| α- tubulin | 1:20000 |  |  | Sigma-Aldrich, ab4074 |
| cCaspase-3 | 1:500 | 1:500 |  | CST, 9664 |
| pHH3 |  | 1:500 |  | CST, 9701 |
| CD34 |  | 1:50 |  | Santa Cruz, sc-18917 |
| CD45R/B220 |  | 1:200 |  | BD Bioscience, 550286 |
| CYR61 |  | 1:2000 |  | Novus, NB100-356 |
| CD4 |  | 1:500 |  | Novus, NBP1-19371 |
| CD8 |  | 1:100 | 1:100 | BD Bioscience, 557668 |
| Ki-67 |  | 1:500 |  | Abcam, ab15580 |
| CD3 |  |  | 1:100 | Proteintech, APC-65077 |
| CD19 |  |  | 1:100 | BioLegend, 115508 |

| **Supplementary table 3**  QuPath scripts for IHC analysis |
| --- |
| **Cleaved caspase-3**  setImageType('BRIGHTFIELD_H_DAB');  setColorDeconvolutionStains('{"Name" : "H-DAB default", "Stain 1" : "Hematoxylin", "Values 1" : "0.65111 0.70119 0.29049 ", "Stain 2" : "DAB", "Values 2" : "0.26917 0.56824 0.77759 ", "Background" : " 255 255 255 "}');  runPlugin('qupath.imagej.detect.cells.PositiveCellDetection', '{"detectionImageBrightfield": "Optical density sum", "requestedPixelSizeMicrons": 0.5, "backgroundRadiusMicrons": 8.0, "medianRadiusMicrons": 0.0, "sigmaMicrons": 1.5, "minAreaMicrons": 10.0, "maxAreaMicrons": 400.0, "threshold": 0.1, "maxBackground": 2.0, "watershedPostProcess": true, "excludeDAB": false, "cellExpansionMicrons": 5.0, "includeNuclei": true, "smoothBoundaries": true, "makeMeasurements": true, "thresholdCompartment": "Nucleus: DAB OD mean", "thresholdPositive1": 0.2, "thresholdPositive2": 0.4, "thresholdPositive3": 0.6000000000000001, "singleThreshold": true}'); |
| **phospho-histone H3**  setImageType('BRIGHTFIELD_H_DAB');  setColorDeconvolutionStains('{"Name" : "H-DAB default", "Stain 1" : "Hematoxylin", "Values 1" : "0.8124 0.52465 0.25446 ", "Stain 2" : "DAB", "Values 2" : "0.32335 0.54612 0.77279 ", "Background" : " 255 255 255 "}');  runPlugin('qupath.imagej.detect.cells.PositiveCellDetection', '{"detectionImageBrightfield": "Optical density sum", "requestedPixelSizeMicrons": 0.5, "backgroundRadiusMicrons": 8.0, "medianRadiusMicrons": 0.0, "sigmaMicrons": 1.5, "minAreaMicrons": 10.0, "maxAreaMicrons": 400.0, "threshold": 0.1, "maxBackground": 2.0, "watershedPostProcess": true, "excludeDAB": false, "cellExpansionMicrons": 5.0, "includeNuclei": true, "smoothBoundaries": true, "makeMeasurements": true, "thresholdCompartment": "Cell: DAB OD mean", "thresholdPositive1": 0.13, "thresholdPositive2": 0.4, "thresholdPositive3": 0.6000000000000001, "singleThreshold": true}'); |
| **CD45R/B220**  setImageType('BRIGHTFIELD_H_DAB');  setColorDeconvolutionStains('{"Name" : "H-DAB default", "Stain 1" : "Hematoxylin", "Values 1" : "0.65711 0.545 0.52076 ", "Stain 2" : "DAB", "Values 2" : "0.4556 0.4556 0.76476 ", "Background" : " 255 255 255 "}');  runPlugin('qupath.imagej.detect.cells.PositiveCellDetection', '{"detectionImageBrightfield": "Optical density sum", "requestedPixelSizeMicrons": 0.5, "backgroundRadiusMicrons": 8.0, "medianRadiusMicrons": 0.0, "sigmaMicrons": 1.4, "minAreaMicrons": 10.0, "maxAreaMicrons": 400.0, "threshold": 0.1, "maxBackground": 2.0, "watershedPostProcess": true, "excludeDAB": false, "cellExpansionMicrons": 1.5, "includeNuclei": false, "smoothBoundaries": true, "makeMeasurements": true, "thresholdCompartment": "Cell: DAB OD mean", "thresholdPositive1": 0.25, "thresholdPositive2": 0.4, "thresholdPositive3": 0.6000000000000001, "singleThreshold": true}'); |
| **CD8**  setImageType('FLUORESCENCE');  runPlugin('qupath.imagej.detect.cells.PositiveCellDetection', '{"detectionImage": "Green", "requestedPixelSizeMicrons": 0.5, "backgroundRadiusMicrons": 8.0, "medianRadiusMicrons": 0.0, "sigmaMicrons": 1.0, "minAreaMicrons": 3.0, "maxAreaMicrons": 50.0, "threshold": 25.0, "watershedPostProcess": true, "cellExpansionMicrons": 2.0, "includeNuclei": true, "smoothBoundaries": true, "makeMeasurements": true, "thresholdCompartment": "Nucleus: Green mean", "thresholdPositive1": 105.0, "thresholdPositive2": 110.0, "thresholdPositive3": 110.0, "singleThreshold": true}');  #Positive threshold was in the range 100 - 120, depending on intensity of staining. |
| **CD4**  setImageType('FLUORESCENCE');  runPlugin('qupath.imagej.detect.cells.PositiveCellDetection', '{"detectionImage": "Green", "requestedPixelSizeMicrons": 0.5, "backgroundRadiusMicrons": 8.0, "medianRadiusMicrons": 0.0, "sigmaMicrons": 1.0, "minAreaMicrons": 3.0, "maxAreaMicrons": 50.0, "threshold": 0.2, "watershedPostProcess": true, "cellExpansionMicrons": 2.0, "includeNuclei": true, "smoothBoundaries": true, "makeMeasurements": true, "thresholdCompartment": "Nucleus: Green mean", "thresholdPositive1": 20.0, "thresholdPositive2": 30.0, "thresholdPositive3": 40.0, "singleThreshold": true}');  #Positive threshold was in the range 15 – 40, depending on intensity of staining. |

| **Supplementary table 4** | | | | | |
| --- | --- | --- | --- | --- | --- |
| Gene name | log2(fc) | q-value | Gene name | log2(fc) | q-value |
| Tgm1 | 6.55 | 0.00 | Il1f9 | 12.00 | 0.00 |
| Dmd | 3.41 | 0.00 | Il1rn | 4.81 | 0.00 |
| Ak1 | -4.75 | 0.00 | Il11ra1 | -2.47 | 0.00 |
| Gper1 | -7.47 | 0.00 | Il13ra2 | 10.94 | 0.00 |
| Grb14 | -7.57 | 0.00 | Il18rap | 2.18 | 0.00 |
| Rgs2 | -4.34 | 0.00 | Il24 | 3.07 | 0.00 |
| Camk2b | -4.77 | 0.00 | Il7 | -1.87 | 0.00 |
| Btg2 | 2.53 | 0.00 | Nacc2 | -1.40 | 0.01 |
| Brsk1 | -2.85 | 0.00 | Cetn4 | -1.67 | 0.01 |
| Gpnmb | -3.75 | 0.00 | Sik1 | 1.13 | 0.01 |
| Bmp7 | -2.30 | 0.00 | Map3k8 | 1.62 | 0.01 |
| Apbb1 | -1.59 | 0.00 | Sfn | 1.84 | 0.01 |
| Atp2b4 | 5.57 | 0.00 | Fzd3 | 1.65 | 0.01 |
| Psrc1 | -2.28 | 0.00 | Cdc25a | 1.22 | 0.01 |
| Piwil4 | -10.66 | 0.00 | Aicda | 4.11 | 0.01 |
| Npr2 | -3.62 | 0.00 | Il12rb1 | -1.86 | 0.01 |
| Spin2c | -3.46 | 0.00 | Il17re | 1.42 | 0.01 |
| Rgs14 | -3.88 | 0.00 | Ccnd2 | -1.72 | 0.01 |
| Ttc28 | -3.01 | 0.00 | Plk2 | 1.08 | 0.02 |
| Ovol2 | 2.28 | 0.00 | Map9 | -9.18 | 0.02 |
| Dnmt3a | 1.54 | 0.00 | Flt3l | -1.28 | 0.02 |
| Ccnb1ip1 | 1.68 | 0.00 | Il3ra | -2.83 | 0.02 |
| Id4 | -2.94 | 0.00 | Mylk2 | 10.10 | 0.03 |
| Tspyl2 | -1.41 | 0.00 | Junb | 1.32 | 0.03 |
| Brdt | -5.71 | 0.00 | Kank2 | -1.07 | 0.03 |
| Xlr | 5.70 | 0.00 | Il23a | 1.10 | 0.03 |
| Atf5 | -2.91 | 0.00 | Il1b | 2.43 | 0.03 |
| Dap | -2.43 | 0.00 | Fbxl21 | -3.26 | 0.04 |
| Il1a | 6.55 | 0.00 | Faddos | 2.12 | 0.05 |
| Il1f6 | 11.71 | 0.00 |  |  |  |

| **Supplementary Table 5** | | | | |
| --- | --- | --- | --- | --- |
| IC_50_ of nitrogen-containing bisphosphonates | | | | |
|  |  | ZOL | ALN | PAM |
| 48h | 4T1 sh-NT | 15.72 ± 0.09 | 19.58 ± 0.08 | 39.7 ± 021 |
|  | 4T1 sh-CD73 | 4.89 ± 0.06^*^ | 6.64 ± 0.05^*^ | 32.6 ± 0.06 |
| 72h | 4T1 sh-NT | 4.25 ± 0.07 | 14.15 ± 0.21 | 32.93 ± 0.03 |
|  | 4T1 sh-CD73 | 1.83 ± 0.33^*^ | 7.8 ± 0.16^*^ | 22.05 ± 0. 08^*^ |

**
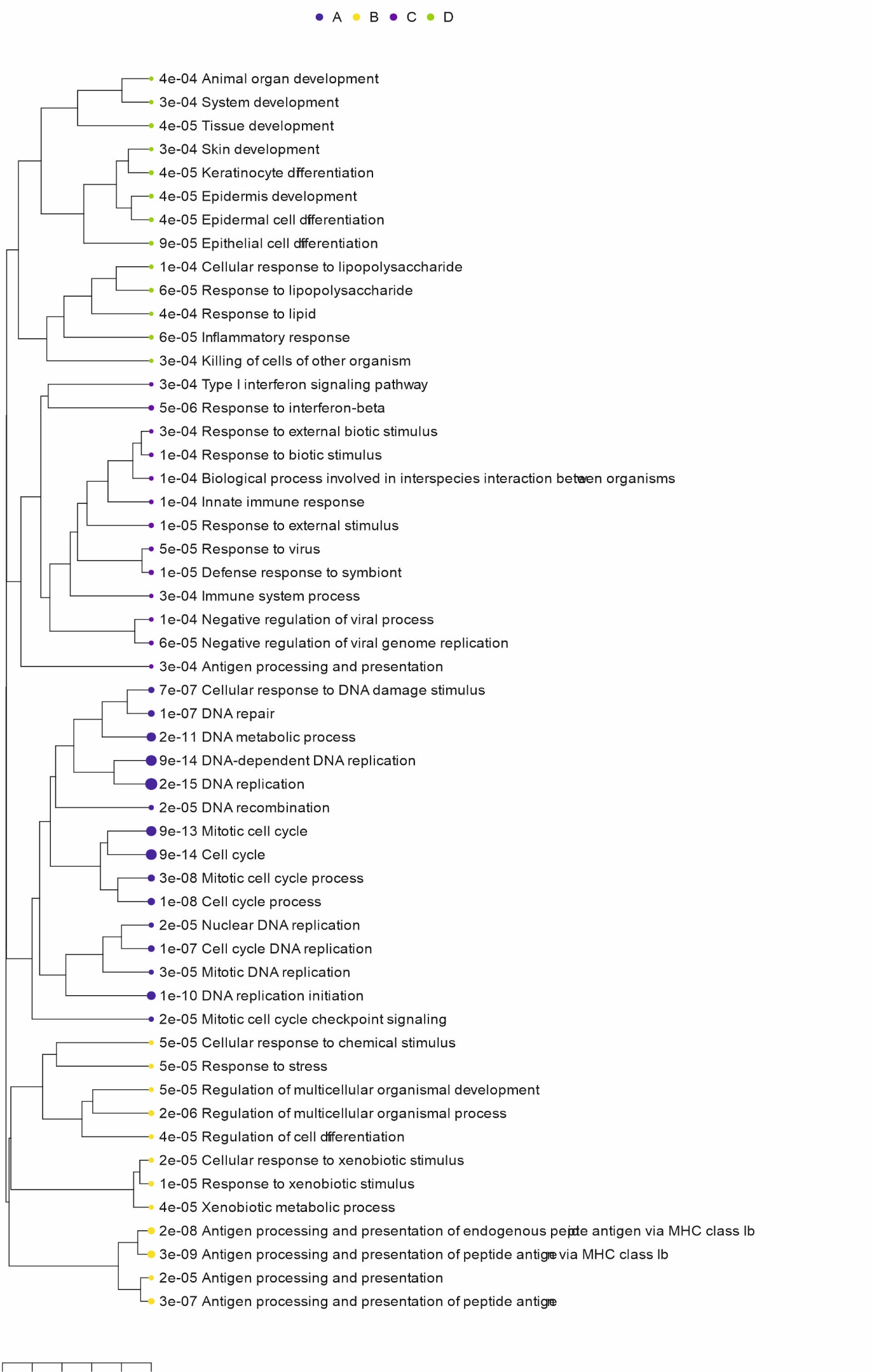
**

**Supplementary figure** **1.** Identification of 4 clusters based on GO Biological Process database in the top 1000 most variable genes from RNA-seq FPKM (fragments per kilobase of exon per million mapped fragments) data in 4T1 sh-CD73 vs 4T1 sh-NT cells. The clusterisation was produced in online available iDEP tool.


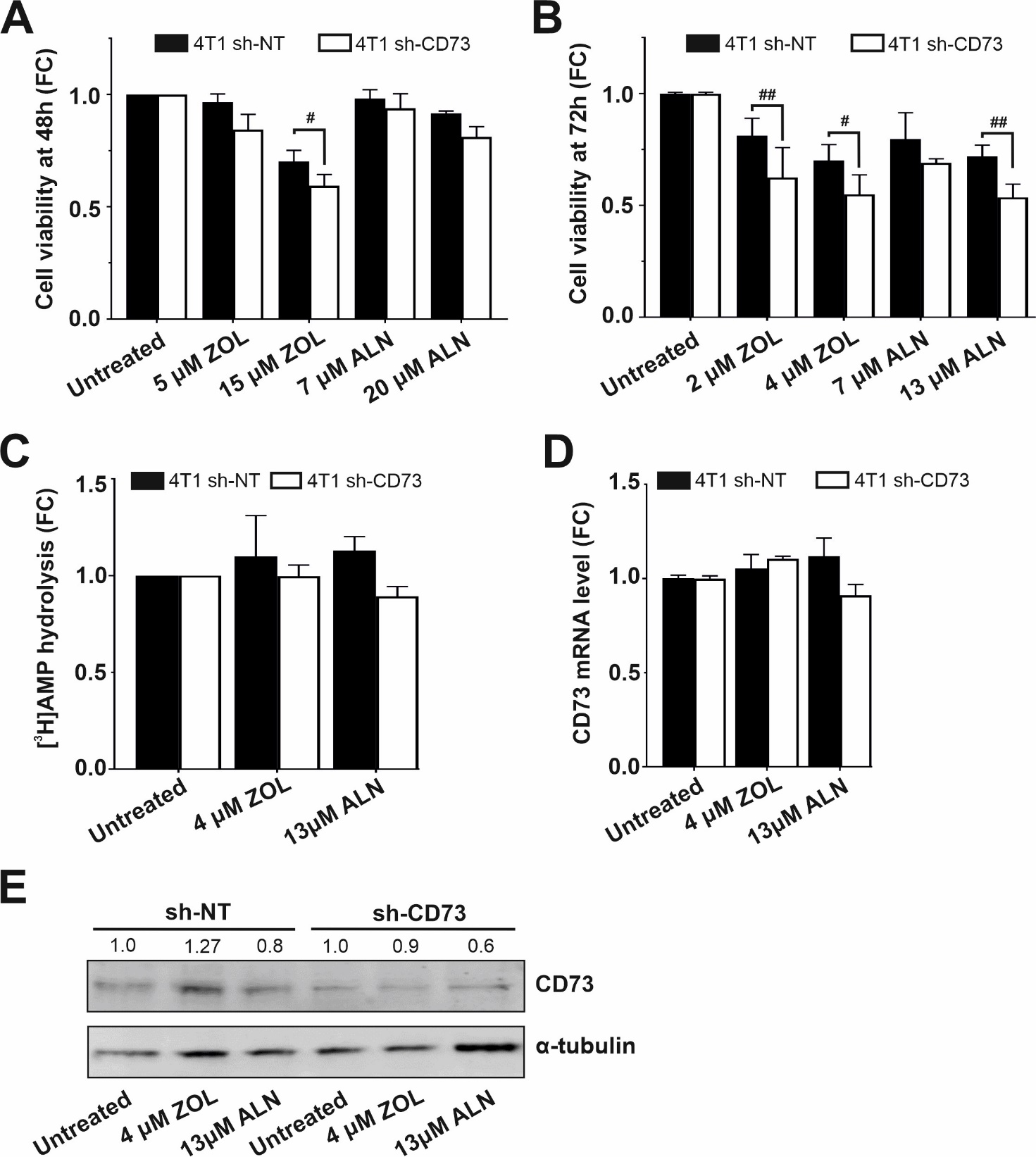


**Supplementary figure 2.** Cell viability of sh-NT and sh-CD73 4T1 cells upon N-BPs treatment during **(A)** 48 h and **(B)** 72 h. Cell viability was measured by WST-8 assays. The bars represent fold-change in viability, as compared with treatment (set to 1). The bars represent mean ± SD, n = 3. (**C**) CD73 activity was determined by TLC as the rate of [^3^H]AMP hydrolysis to [^3^H]adenosine in 4T1 sh-NT and sh-CD73 cells. The results are expressed as a fold change of [3H]AMP-hydrolyzing activity determined in sh-NT cells (mean ± SD, n=3). (**D**) CD73 mRNA expression was analyzed by qPCR. The bars represent CD73 mRNA relative to the housekeeping gene TBP, data is expressed as mean ± SD, n = 3. (**E**) CD73 protein expression was analyzed by Western blotting. # P<0.05 and ## P<0.001 are considered to be statistically significant between 4T1 sh-CD73 and sh-NT cells, by two-tailed Student’s *t –* test.

**
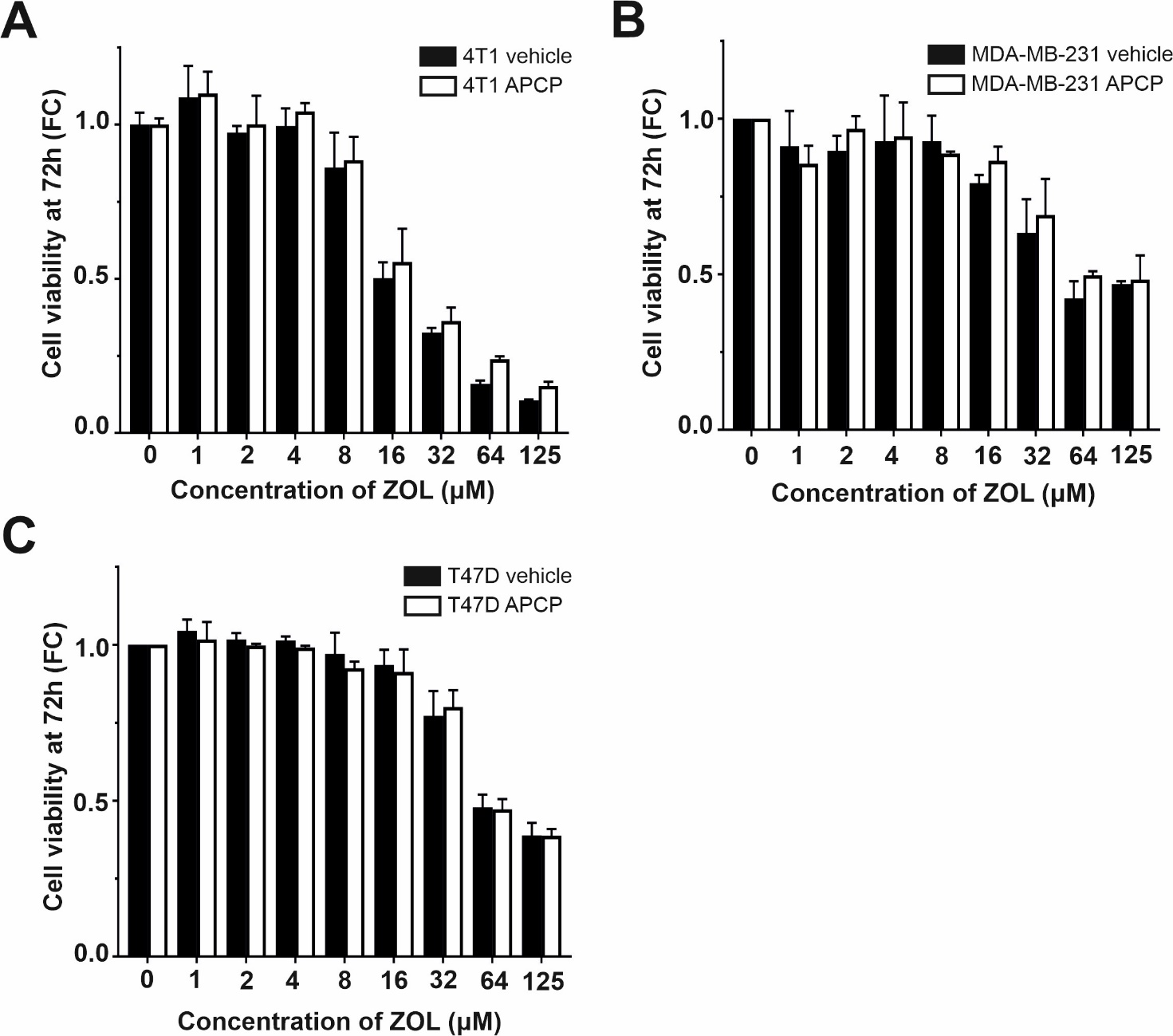
**

**Supplementary figure 3.** (**A**) Viability of APCP- or vehicle-treated 4T1, (**B**) MDA-MB-231 cells, (**C**) T47D cells upon zoledronate treatment, were measured by WST-8 assays. The bars represent fold-change, set to 1. The bars represent mean ± SD, n = 3.

**
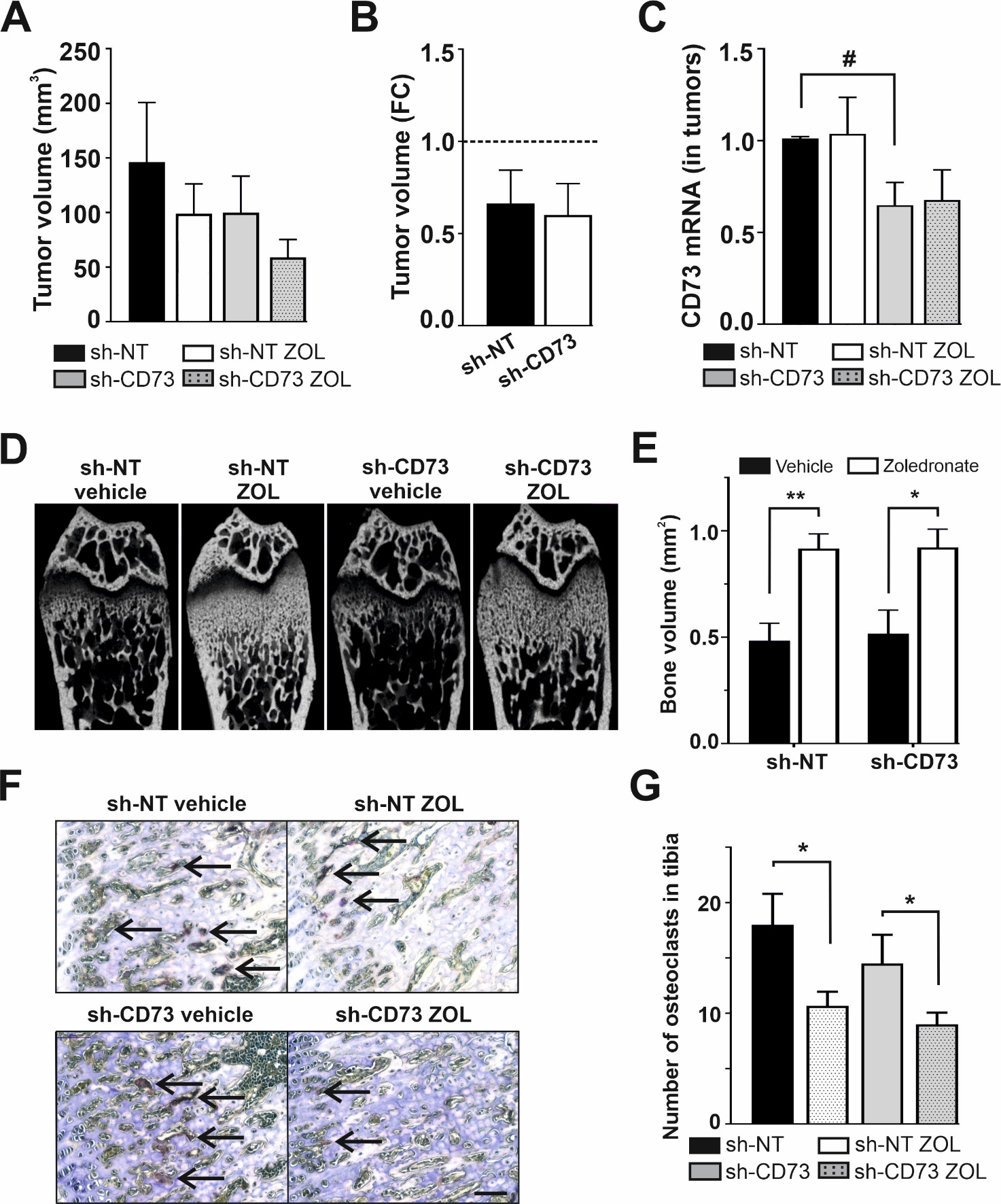
**

**Supplementary figure 4. (A)** Tumor volume at the sacrifice. **(B)** Fold-change of tumor volume at the sacrifice. **(C)** CD73 mRNA expression in tumors. **(D)** Representative images of tibia. **(E)** Bone volume**. (F)** Representative images of TRAcP staining of tibia. Scale bar = 50µm. **(G)** Number of osteoclasts below growth plate per area in tibia. * P < 0.05; ** P < 0.01; # P < 0.05 are considered to be statistically significant compared to the respective controls, by one-way ANOVA with a Sidak’s post-test.

**
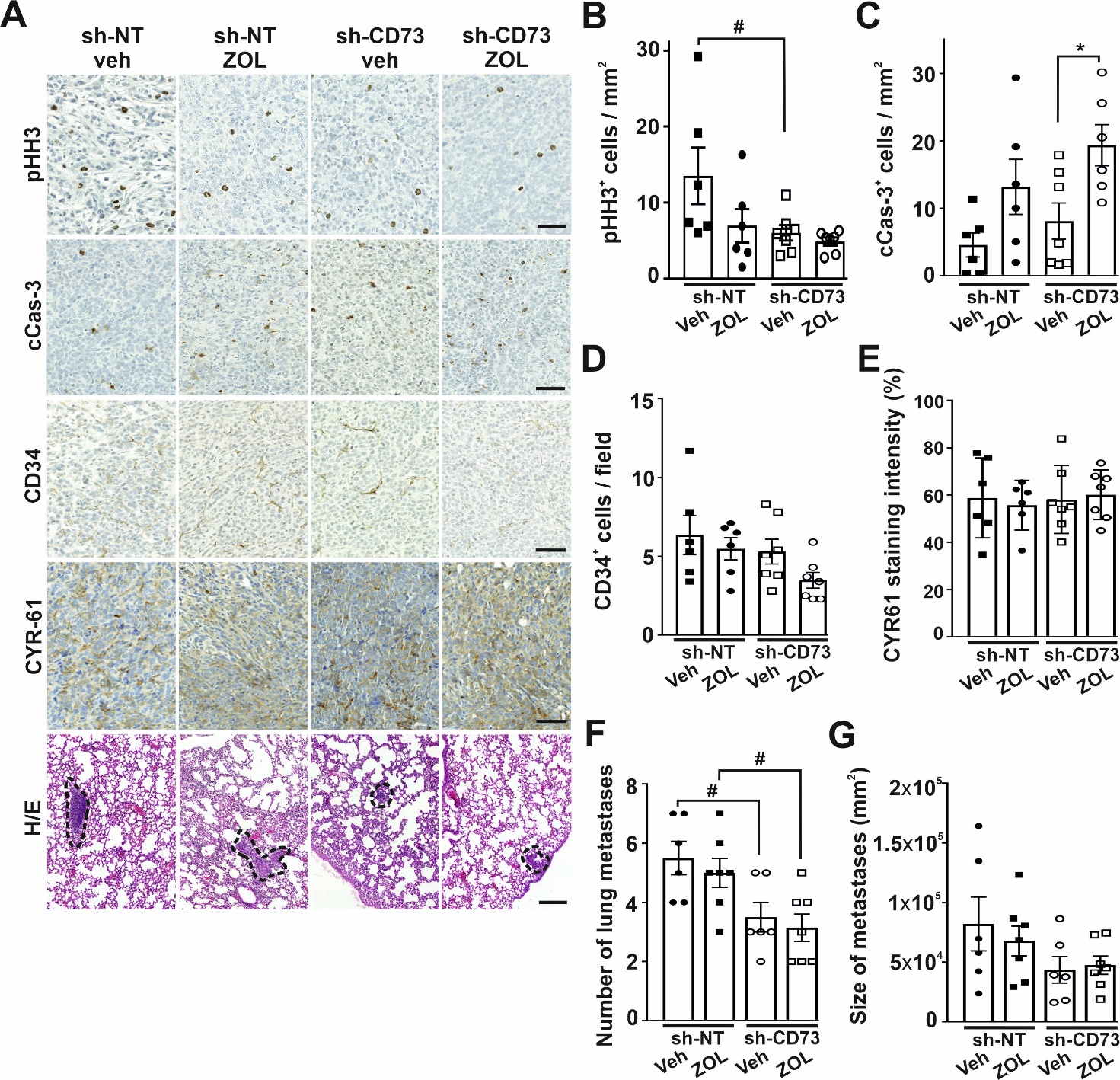
**

**Supplementary figure 5. (A)** Representative images of staining in sh-NT and sh-CD73 tumors. Scale bar 100 μm. Representative images of stained lungs. Scale bar 500 μm. Number of **(B)** pHH3-positive cells, **(C)** cCas-3-positive cells, **(D)** CD34-positive and **(E)** CYR-61-positive cells from 4T1 sh-NT and sh-CD73 tumors. Number **(F)** and size **(G)** of lung metastases. Data is expressed as mean ± SEM, by a two-tailed Student’s t – test. * P < 0.05, comparing within the same group upon different treatment; # P < 0.05, comparing sh-CD73 treated tumors vs. sh-NT treated tumors.

**
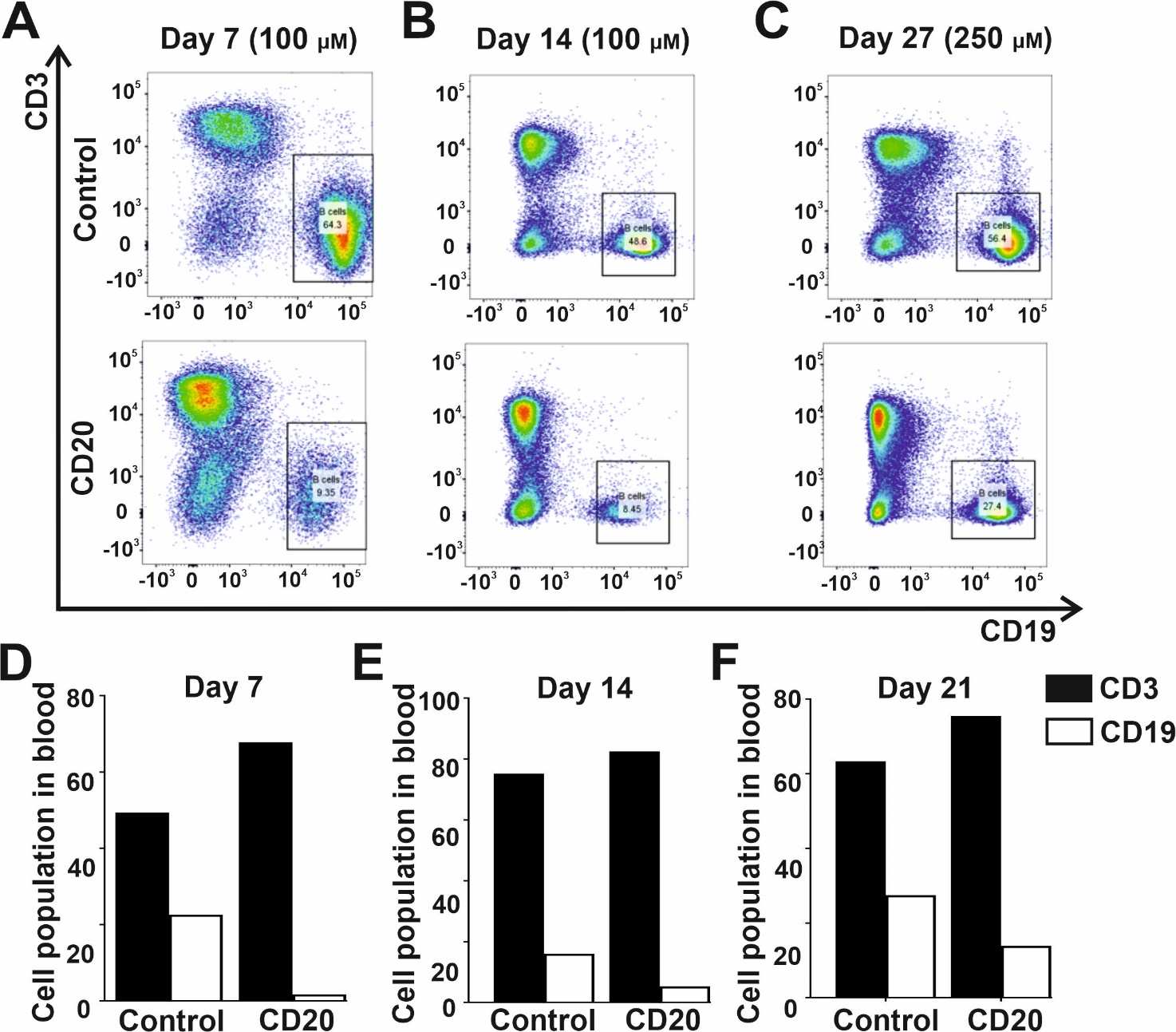
**

**Supplementary figure 6. (A)** Representative images of dot plots of splenocyte flow cytometry analysis upon control IgG and anti-CD20 treatment. Number of CD3- and CD19-positive cells on **(B)** day 7, **(C)** on day 14, **(D)** on day 21.
